# Amivantamab, the first epidermal growth factor receptor and mesenchymal–epithelial transition factor inhibitor, associated with penile and inguinal ulcers and paronychia

**DOI:** 10.1016/j.jdcr.2024.01.014

**Published:** 2024-01-27

**Authors:** Karina J. Cancel-Artau, Angélica C. Marrero-Pérez, Eduardo A. Michelén-Gómez, Xavier Sánchez-Flores

**Affiliations:** a Department of Dermatology, University of Puerto Rico School of Medicine, San Juan, Puerto Rico; b University of Medicine and Health Sciences, Basseterre, Saint Kitts and Nevis

**Keywords:** amivantamab, endothelial growth factor receptor inhibitor, ulcer

## Introduction

Amivantamab is a monoclonal antibody targeted against the epidermal growth factor receptor (EGFR) and mesenchymal–epithelial transition factor (MET) receptor.^1^ Approved recently, on May 21, 2021, for non–small cell lung cancer.^2^ Cutaneous side effects are similar to those commonly observed with EGFR inhibitors, including papulopustular lesions, paronychia, and pruritus.^3^ The EGFR protein is essential for skin growth and skin barrier integrity, which reflects the cutaneous adverse effects observed with EGFR inhibitors.^3^ We report a case of genital ulcers and paronychia following the administration of amivantamab.

## Case Presentation

**Fig 1:**
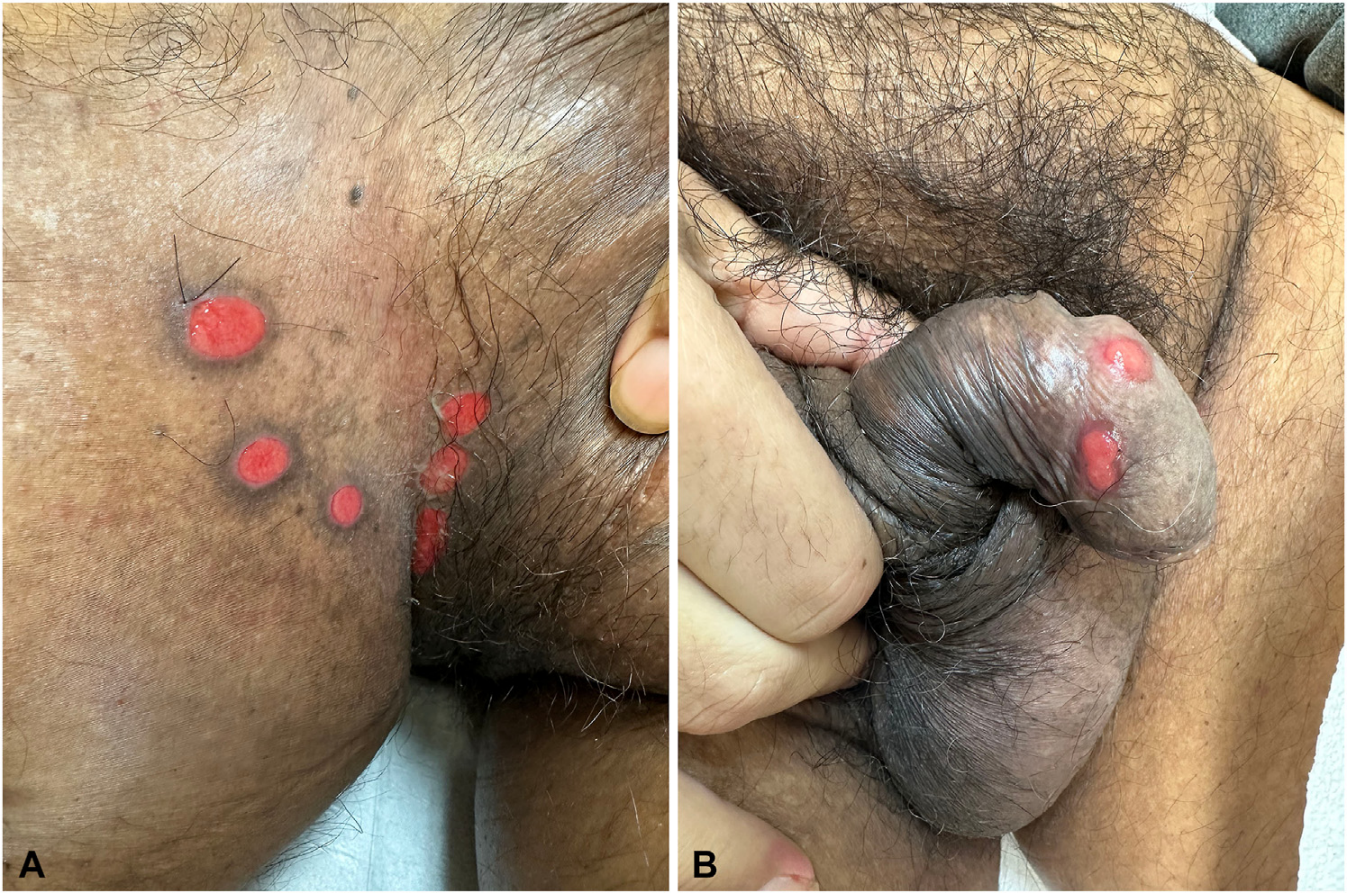
Several oval ulcers with scalloped borders on the (**A**) right inguinal fold and (**B**) penile shaft.

**Fig 2:**
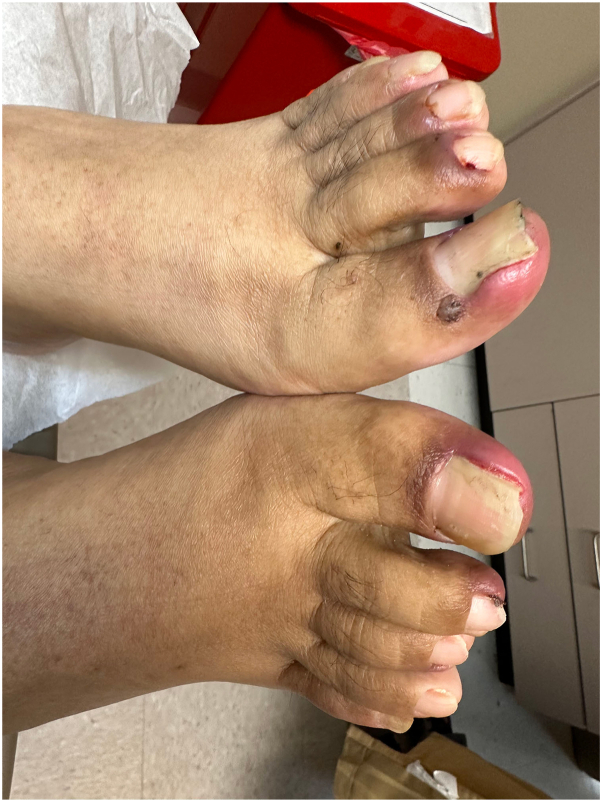
Erythema and swelling of the proximal nail folds with hemorrhagic crust on the first, second, and third toes on both feet.

**Fig 3:**
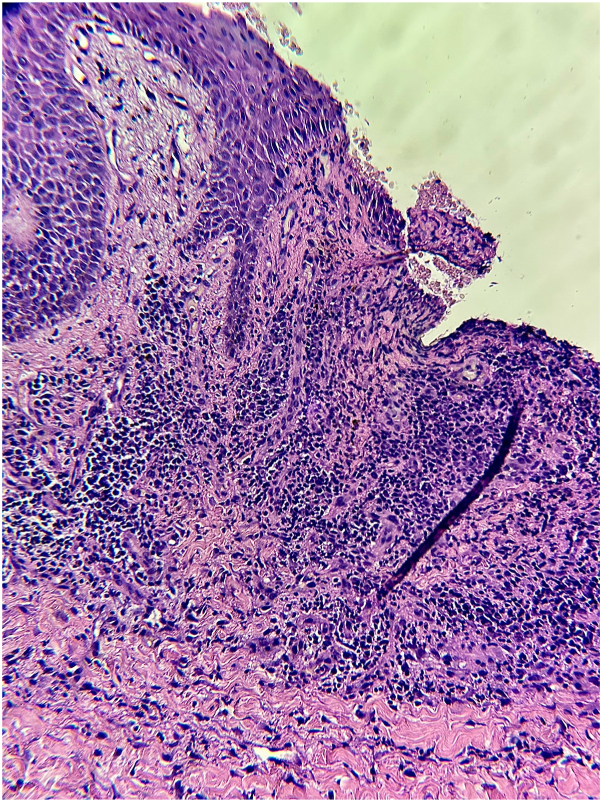
A skin biopsy specimen from an active lesion on the right inguinal fold revealed an ulcer with lymphoplasmacytic infiltrate.

A 57-year-old man with a history of advanced non–small cell lung cancer with distant metastasis presented to the clinic with painful lesions in the inguinal folds and toenails. Six weeks prior to the visit, amivantamab was started. At the time of presentation, he had already received 5 infusions. Two weeks after starting treatment, he developed small ulcers in the groin and swollen toenails. After each subsequent infusion, new ulcers appeared in the same sites and the penile area. Other associated symptoms included cracked lips and dry mouth. Prior to the initial visit, lesions had been treated with potent topical steroids and antifungals with minimal response. The patient denied having any family members with similar lesions, previous episodes of similar lesions, or systemic symptoms. Physical examination revealed multiple, tender, oval ulcers with scalloped borders on bilateral inguinal folds and penile shaft (Fig 1). There was erythema and swelling with an underlying hemorrhagic crust on the proximal nailfolds of the first, second, and third toes bilaterally (Fig 2). No inguinal lymphadenopathy was identified. Laboratory study results were negative for Epstein–Barr virus IgG and immunoglobulin M, cytomegalovirus, HIV, herpes simplex virus type 1 and 2 IgG and immunoglobulin M, rapid plasma reagin test, and Venereal Disease Research Laboratory test. A skin biopsy specimen from an active lesion on the groin showed an ulcer with a lymphoplasmacytic infiltrate (Fig 3). Based on the clinical presentation, we suspected the possibility of drug-related toxicity, and amivantamab was held for 1 month. During this interval, paronychia resolved with clobetasol and mupirocin cream, and the ulcers showed significant improvement with re-epithelization. However, due to the patient’s malignancy, the oncologist resumed amivantamab. After subsequent infusion, there was a recurrence of ulcers in the same sites, and perianal erythema. To rule out chancroid ulcers, the patient completed a 3-week course of azithromycin, and lesions persisted. Therefore, a diagnosis of amivantamab-induced inguinal and penile ulcers and paronychia was established.

## Discussion

The EGFR protein is essential to maintain epidermal homeostasis.^3^ When its function is disrupted, it can adversely affect the skin. PRIDE complex refers to the classic cutaneous side effects observed with EGFR inhibitors, including papulopustular rash, paronychia, hair growth abnormalities, itching, and dryness.^4^ Amivantamab is the first EGFR and MET inhibitor target therapy for locally advanced or metastatic non–small cell lung cancer with EGFR exon 20 insertion mutations.^1^^,^^5^ In the CHRYSALIS phase 1 clinical trial, cutaneous lesions were the most common adverse reaction.^1^ Skin lesions were consistent with those seen with other EGFR inhibitors, including acneiform eruptions, paronychia, and stomatitis.^1^

Conversely, the toxicity profile associated with MET inhibition is unrelated to the skin, including lower extremity edema, gastrointestinal distress, fatigue, and transaminitis, none of which were observed in our patient.^5^

The abrupt onset of lesions and temporal resolution following drug initiation and withdrawal, and concomitant paronychia and dryness commonly observed with EGFR inhibitors, support the diagnosis of EGFR inhibitor cutaneous toxicity. The EGFR is present in basal epidermal keratinocytes, and studies have shown that inhibition of this receptor induces cell cycle growth arrest and terminal differentiation.^6^^,^^7^ Skin changes such as epidermal atrophy, hyperplasia, and abnormal differentiation observed in various skin diseases may be mediated through disruption of the EGFR signaling cascade.^7^ Histologic changes during therapy include thinning of the corneal layer and granular layer, vacuolar degeneration of the basal layer, and apoptotic keratinocytes.^8^ These molecular and histologic changes may result in skin necrosis and ulceration, as seen in our patient.

Although the patient did not exhibit all of the adverse events enclosed within the PRIDE complex, he experienced 3, including dry mouth, cracked lips, and paronychia. To our knowledge, a single case of skin ulcers appearing after initiating an EGFR inhibitor has been reported.^9^ The patient developed an ulcer on the palm of the hand 2 weeks after initiating therapy with gefitinib, another commonly used EGFR inhibitor for lung cancer. Therapy was interrupted for 2 weeks, and the patient was treated with topical steroids and antibiotics, with resolution of the ulcer. Our patient responded inadequately to topical steroids while on treatment with amivantamab. However, after discontinuing amivantamab, the lesions responded positively with re-epithelialization.

After a literature review, apart from clinical trials, a single case of amivantamab-induced adverse cutaneous reactions was found.^10^ A 53-year-old man developed facial, scalp, buttocks, and lower limb lesions after 3 weeks of treatment. Notably, the patient developed erosive erythema in the perianal region, erythematous papules on the scrotum, and paronychia-like changes, similar to our patient. Therapy was continued with lesions recurring following each infusion. However, lesions improved with kangfuxin liquid, compound polymyxin B ointment, and recombinant human epidermal growth factor gel.

The differential diagnosis of genital ulcers in an immunocompromised patient includes chancroid, syphilis, cytomegalovirus, Behçet disease, lymphogranuloma venereum, and herpes simplex virus. The absence of viral cytopathic changes on histology makes a viral infection less likely. However, given the unavailability of polymerase chain reaction results in our case, the possibility of herpes zoster virus cannot be entirely excluded. Negative infectious workup, nonspecific histologic findings, unresponsiveness to azithromycin, presence of paronychia, dryness, and temporal relationship with drug ingestion support the diagnosis of EGFR-inhibitor cutaneous toxicity. Among other differential diagnoses, the possibility of monkeypox in immunocompromised patients presenting with genital ulcers cannot be overlooked, which has resurged in the past year and a half.^11^

Herein, we present a case of penile and inguinal ulcers and paronychia following amivantamab administration. Although rare, health care providers should be aware of genital ulcers as a potential cutaneous side effect associated with amivantamab and possibly other EGFR and MET targeting therapies and consider prompt interruption of therapy, if feasible.

## Conflicts of interest

None disclosed.
